# Gd^3+^ and Calcium Sensitive, Sodium Leak Currents Are Features of Weak Membrane-Glass Seals in Patch Clamp Recordings

**DOI:** 10.1371/journal.pone.0098808

**Published:** 2014-06-19

**Authors:** Adrienne N. Boone, Adriano Senatore, Jean Chemin, Arnaud Monteil, J. David Spafford

**Affiliations:** 1 Department of Biology, University of Waterloo, Waterloo, Canada; 2 CNRS, UMR-5203, Institut de Génomique Fonctionnelle, INSERM, U661, Universités de Montpellier 1 & 2, UMR-5203, Montpellier, France; Dalhousie University, Canada

## Abstract

The properties of leaky patch currents in whole cell recording of HEK-293T cells were examined as a means to separate these control currents from expressed sodium and calcium leak channel currents from snail NALCN leak channels possessing both sodium (EKEE) and calcium (EEEE) selectivity filters. Leak currents were generated by the weakening of gigaohm patch seals by artificial membrane rupture using the ZAP function on the patch clamp amplifier. Surprisingly, we found that leak currents generated from the weakened membrane/glass seal can be surprisingly stable and exhibit behavior that is consistent with a sodium leak current derived from an expressible channel. Leaky patch currents differing by 10 fold in size were similarly reduced in size when external sodium ions were replaced with the large monovalent ion NMDG^+^. Leaky patch currents increased when external Ca^2+^ (1.2 mM) was lowered to 0.1 mM and were inhibited (>40% to >90%) with 10 µM Gd^3+^, 100 µM La^3+^, 1 mM Co^2+^ or 1 mM Cd^2+^. Leaky patch currents were relatively insensitive (<30%) to 1 mM Ni^2+^ and exhibited a variable amount of block with 1 mM verapamil and were insensitive to 100 µM mibefradil or 100 µM nifedipine. We hypothesize that the rapid changes in leak current size in response to changing external cations or drugs relates to their influences on the membrane seal adherence and the electro-osmotic flow of mobile cations channeling in crevices of a particular pore size in the interface between the negatively charged patch electrode and the lipid membrane. Observed sodium leak conductance currents in weak patch seals are reproducible between the electrode glass interface with cell membranes, artificial lipid or Sylgard rubber.

## Introduction

Ion channel or receptor genes are routinely cloned from tissue mRNA and expressed in surrogate cells such as human HEK-293T cell lines for detailed functional analyses using patch clamp recording [1]. “HEK” in HEK-293T stands for Human Embryonic Kidney cell, which is likely a misnomer, since the cell line has properties of immature neurons and likely was a cell derived from a neuronal lineage [2]. “293” was the product of the 293^rd^ adenoviral transformation, carried out by Frank Graham in van der Eb’s lab in the early 1970s [3]. “T” stands for the incorporated SV40 Large T-antigen that allows for episomal plasmid replication within the host cell line for long term, expression of desired genes after a single transient transfection of plasmids containing ion channel or receptor genes. Levels of expression can vary widely with transiently transfected genes, and some difficult to express genes produce small, elusive currents, even after long incubation periods. Normal expression efficiency of some ion channel or receptor genes can be uncomfortably close to background levels and abundantly expressing cells can be lost, due to cell death from the toxicity of over-expressed genes under strong mammalian viral promoters. Assessment of the expressed channel is assayed in relation to known background currents, including native currents and the ion currents generated through leaky patch clamp seals. Native Na^+^, K^+^ and Ca^2+^ conductance channel currents in HEK-293T cells are known and can be accounted for [4]–[10]. A leaky patch clamp seal current is a technical artifact of patch clamp recording in whole cell mode caused by an alternative low resistance pathway for ions to travel to the ground state instead of passing through membrane channels [11]. Leaky patch conductances can be subtracted, off- or on-line, as non-selective, ohmic linear conductances [12]. Small contaminating leaks can often be ignored assuming that the leak is a small, linear, ohmic component of the aggregate current waveform.

We became interested in defining the properties of the leaky patch currents, in order to form a baseline from which we could separate the total current from sodium leak conductances that we expected to generate from expressible ion channels, from unique snail NALCN clones that we had identified [13], [14]. Snail NALCN like those of many other protostome invertebrates, have alternatively spliced ion selectivity filters that resembled both calcium channels (EEEE) and sodium leak channels (EKEE or EEKE), so we were expecting to be able to measure NALCN channel activity with differing ion selectivities above the background leak conductance due to weakened membrane patch seals. We also wanted to confirm the expression features of the Gd^3+^ and calcium sensitive mammalian sodium leak conductance currents reported for the mammalian NALCN cDNA transfected in HEK-293T cells [15]–[18].

Intuitively, we had envisioned leaky patch currents as current arising from ion flow in saline between the pipette glass and the cell membrane, where tighter seals have narrower gaps [19]. We found, however, that these nominally artifactual currents could be inhibited by replacing Na^+^ with the large monovalent ion, NMDG^+^. The leaky patch current were potentiated in lower external calcium concentration and were inhibited with low concentrations of Gd^3+^ (10 µM). The exhibited a low sensitivity to Ni^2+^ suggesting ion selectivity in spite of leak currents that varied tenfold in amplitude. We have dubbed this ionic current, I**_L_**_eaky **P**atch_ or **I_LP_**. **I_LP_** has the reproducible features of an ion channel current but which appears to arise from cation selectivity of the glass-membrane seal interface [20]. Currents through an ion selective patch seal can confuse interpretations of currents from expressible channels [15]–[18].

## Materials and Methods

### Culturing of HEK-293T Cells

Mammalian cells (HEK-293T) were manipulated as previously described [13], [21]–[24] and we have outlined our procedure for culturing and maintaining HEK-293T cell lines, and optimized recording of ion channels in HEK-293T cells via a video journal (JoVE) article [25]. Fully confluent HEK-293T cells in a 6 mL vented flask were detached using warm Trypsin (Sigma-Aldrich) and split 1∶4 into 35 mm culture dishes containing Dulbecco’s Modified Eagle’s Medium (DMEM) supplemented with 10% fetal bovine serum (FBS; Sigma) and 100 µM sodium pyruvate (Sigma-Aldrich). After over-night incubation at 37°C to permit cell adhesion and recovery, cells were incubated at 37°C for 4–6 hours, washed 1× with warm supplemented DMEM containing penicillin/streptomycin (Sigma-Aldrich; as per manufacturer), and transferred to 28°C. After 1–2 days, cells were detached by trypsinization (as above). 0.5 ml of cell suspension was transferred to a 60 mm culture dish containing 8 uncoated glass coverslips (Circles No. 1–0.13 to 0.17 mm thick; Size: 12 mm) and 4.5 ml media without FBS, resulting in a reduction of the total media FBS concentration to 1%. Lowered FBS concentrations created more sickly HEK-293T cells that were more prone to generate leaky patch currents, but low FBS concentrations weren’t required to generate leaky patch currents. Culture dishes were incubated at 37°C for 4 hrs and then transferred to a 28°C, 5% CO_2_ incubator for 2–3 days before electrophysiology experiments were carried out.

### Electrophysiological Recording of Leaky Patch Current (I_LP_)

We formed tight gigaseals with polished pipettes on control HEK-293T cells, in the whole cell configuration generated by strong suction from the cell-attached mode. Whole-cell patches with tight gigaseals possessed no obvious currents, suggesting nominal conductances through native ion channels in the control HEK-293T cells we were recording. We then artificially weakened the gigaohm patch seals by voltage pulses using the ZAP function on the AxoPatch 200B or MultiClamp 700B amplifier for 500 us, 1 ms, 2 ms or 5 ms, to facilitate the generation of leak currents. The extent of zapping required varied cell-to-cell. Gigaseals often reformed after zapping, and cells then repeatedly zapped until a stable leak was formed. The most stable, consistent leak currents were in the −100 pA to −200 pA range generated at a voltage of −100 mV. The leak current was defined as the linear part of the current-voltage relationship in a voltage ramp generated for one second from −100 mv and ending at +100 mV. The voltage ramp was repeated every 10 seconds to generate a sweep. Leak currents sizes was measured as the inward current amplitude at −100 mV and the outward current amplitude at +100 mV.

External bath solution for whole cell patches included (in mM; all chemicals from Sigma): 150 NaCl, 3.5 KCl, 1 MgCl_2_, 1.2 CaCl_2_, 20 glucose, and 10 HEPES (pH 7.4 with NaOH) with a measured osmolarity of ∼320 mOsm/L. For replacement of external sodium, 150 mM NaCl was replaced with 150 mM large monovalent ion, N-methyl-D-glucamine (NMDG^+^), and the pH was adjusted with HCl and the osmolarity measured at ∼320 mOsm/L. Stock solutions of mibefradil and verapamil were prepared in 0.01% DMSO. Divalent cations, verapamil and mibefradil were freshly diluted in external solution just prior all experiments. The internal solution contained (in mM): 150 Cs^+^, 120 methanesulfate, 10 NaCl, 10 EGTA, 4 CaCl_2_, 0.3 Na_2_-GTP, 2 Mg-ATP, and 10 HEPES pH 7.4 with CsOH (∼300 mOsm/L). HEK-293T cells were recorded by the whole-cell patch clamp method using an AxoPatch 200B or MultiClamp 700B amplifier from Molecular Devices, combined with their Digidata 1440A Data Acquisition System and pCLAMP 10 Software. Recordings were carried out at room temperature, with patch pipettes bearing resistances between 2 to 5 MΩ.

HEK-293T cells were perfused in a constant stream of solutions with a ValveLink8 pinch valve perfusion system (Automate Scientific). Perfusion solutions contained NMDG^+^ or low 0.1 mM [Ca^2+^]_ex_. We were concerned of pressure effects of perfusion on the integrity of the leak current and possible contribution of mechanosensitive channels, so drug additions were sometimes carried out by injecting doses of 25x concentrated drug to the 2880 ul dish in 6×20 uL aliquots, so that a 1x final concentration of drug bathed cells in the dish without significant mechanical disturbance. We waited for many minutes to be certain that adequate mixing of the drug concentration in the dish was achieved.

## Results

**Figure 1:**
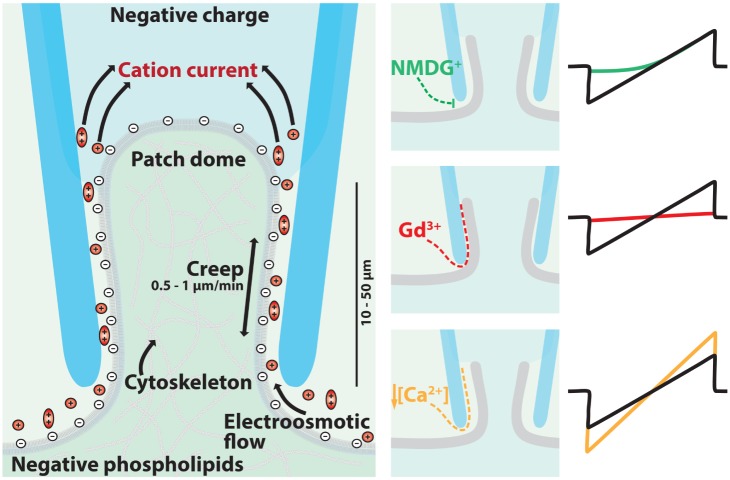
Ionic currents generated through a leaky patch (I_LP_) generates a sodium leak conductance which is potentiated in low external calcium and is highly sensitive to Gd^3+^ block. Left: Architecture of the cell attached patch as detailed by Fredrick Sachs [20]. The membrane seal is the 10–50 micron of adherent membrane scaled along the inside walls of the glass pipette. The negatively charged glass and negatively-charged membrane generates a seal pocket that is filled with a lubricated layer of cations. The electro-osmotic flow of mobile cations in the seal space is the motor driving membrane patch creep. Right: Extracellular treatments that regulate whole cell leak currents in the patch seal. Less mobile cations like NMDG^+^ slows the inward electro-osmotic flow of ions compared to Na^+^ ions. Gd^3+^ has a high local charge density that titrates positive charges into the patch seal space, neutralizing the electrostatic repulsion of the negatively charged glass electrode and membrane, improving the seal resistance, and causing a decrease in leak current size. Lowering Ca^2+^ reduces the adhesive force between the glass and membrane and causes increases in leak current sizes.

We formed whole cell patches with tight Gigaohm seals without observable currents that would result from native conductances in HEK-293T cells. Leaky patch seals currents (**I_LP_**) with a weaker membrane seal (eg. low Mega-ohm range of membrane resistance), were generated by artificial membrane rupture by voltage pulse (ZAP function) on the patch clamp amplifier (Molecular Devices), to avoid a possibility of ascribing leaky patch seal currents to possible contaminating ion channel currents native to HEK-293T cells. These recordings with weaker membrane patch seals can remain stable and unchanging in their leak current size for a length of a standard recording session, from minutes to an hour (**Figure 1**).

**Figure 2:**
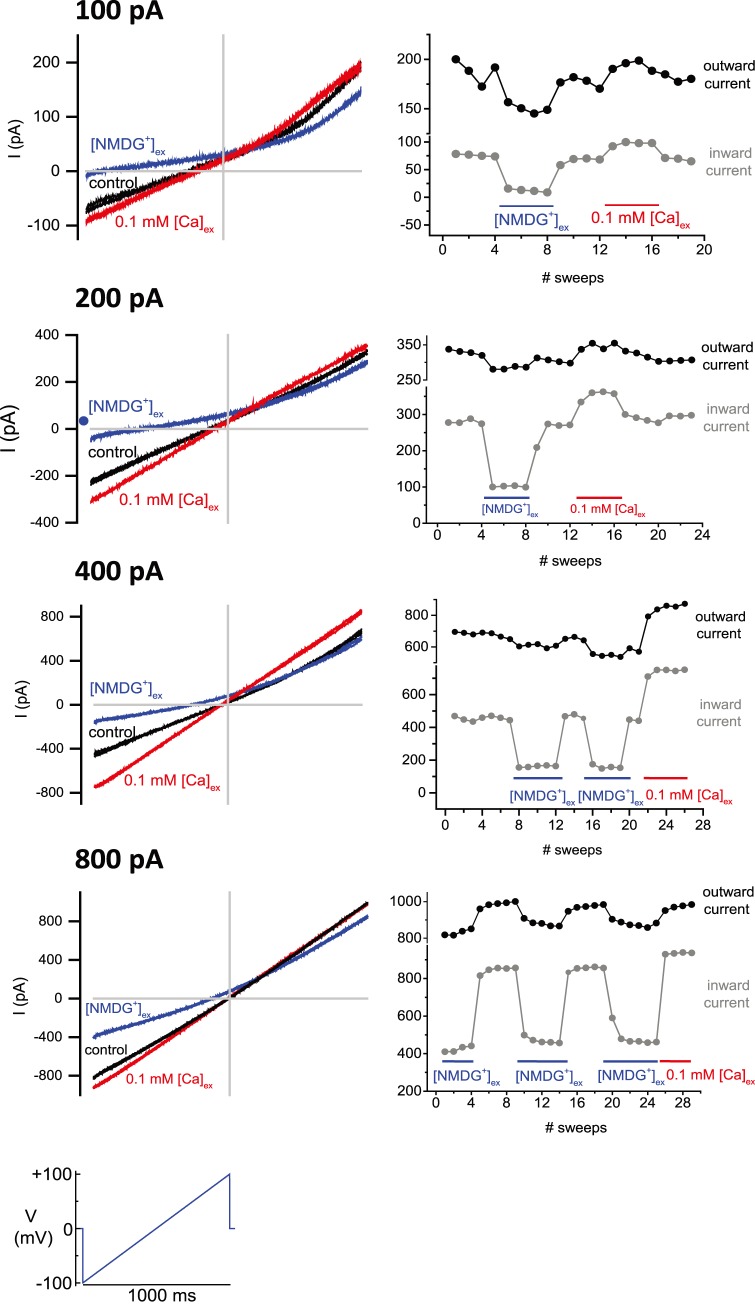
Voltage ramp recordings of leaky patch current (I_LP_) illustrating the stable sodium leak currents of differing sizes (approx. 100 pA, 200 pA, 400 pA, 800 pA) and their consistent behavior in successive sweeps (every 10 seconds) in response to perfusion. (A) Representative tracings. Axes cross at zero. X = voltage (mV), Y = current (pA). (B) Size of outward currents were measured at +100 mV and the size of inward currents measured at −100 mV in the ramp protocol. There is a dramatic decrease in I_LP_ currents after perfusion of (1.2 mM [Ca]_ex_, 150 mM [NMDG^+^]_ex_), and an increase in I_LP_ currents after (0.1 mM [Ca]_ex_, 150 mM [Na^+^]_ex_), compared to perfusion of control external solution (1.2 mM [Ca]_ex_, 150 mM [Na]_ex_).

We used standard conditions for generating non-selective cation leak currents with extracellular solutions that contained 150 mM Na^+^, 3.5 mM K^+^ and 1.2 mM Ca^2+^ and intracellular solutions containing 150 mM Cs^+^, 10 mM Na^+^, 4 mM Ca^2+^ and 10 mM EGTA. The presentation of **I_LP_** is an ohmic linear current with variable rectification, but often has no rectification (see **Figure 2** for representative examples). It is possible that observed rectification at very positive potentials was due to contribution of endogenous ion channels as part of the “leak current”. Leak currents change proportionally in current size in response to a voltage ramp from −110 mV to +100 mV, reversing direction from the hyperpolarizing to depolarizing direction with an ordinate value normally near zero (**Figure 2**). Perfusion of extracellular solutions increases or decreases the slope of the linear leak conductance in a rapid and reversible manner (illustrated in **Figure 2**).

**Figure 3:**
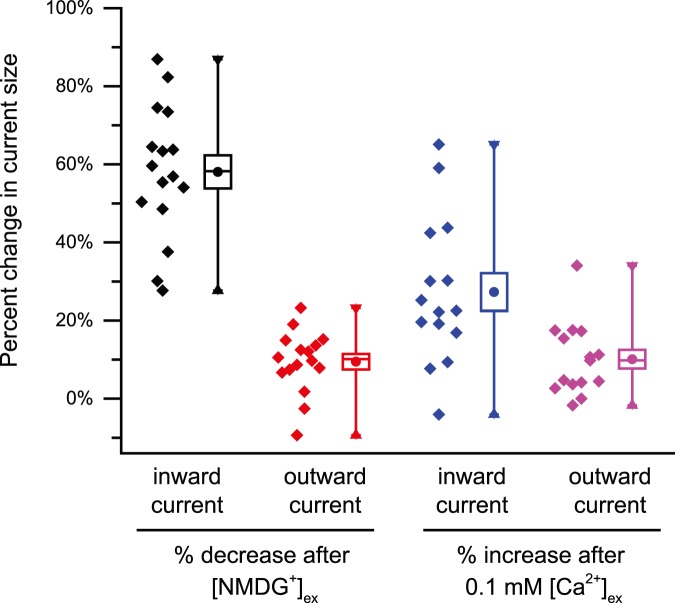
Percent change in current size of leaky patch current (I_LP_) inward current (measured at −100 mV in ramp protocol) and I_LP_ outward current (measured at +100 mV in ramp protocol) in response to NMDG replacement of external Na ions or 0.1 mM Ca replacement of 1.2 mM external calcium. Data points (left) and box plot (mean +/− SEM, right). Note that the relative change is remarkably consistent despite the dramatic difference in current densities of I_LP_ ranging from 0.38 to 171.28 pA/pF, with average 36.70+/−2.93 in 18 HEK-293T cells.

We chose HEK-293T cells with significant but stable **I_LP_** currents, typically in the range of 100–300 pA but these can be stable as large as 1 nA, measured at a holding potential voltage of −110 mV. Inward currents through **I_LP_** is largely due to inward Na^+^ influx, because inward currents through **I_LP_** were mostly lost when equimolar membrane impermeant NMDG^+^ replaces 150 mM Na^+^ in the external solution (**Figure 3**). There was a 58.3% +/−4.3 reduction in total inward leak current size, ranging from 27% to 88%, n = 16 when NMDG^+^ replaced Na^+^ in external solution. I_LP_ are characterized as non-specific currents, so outward currents could be carried by the major internal ion, Cs^+^, which is not as affected by the changing external conditions as inward currents were (**Figure 3**). Perceived outward current flux could in theory, be supported by an influx of Cl^−^ anions too. Lowering the standard external calcium concentrations of 1.2 mM to 0.1 mM, increased inward I_LP_ currents by an average of 27.3% +/−4.8 in leak current size, ranging from <0% to 65%, n = 15) (**Figure 3**). The responses in leak conductances to external NMDG^+^ and 0.1 mM calcium was remarkably consistent in spite of the vastly differing sized leak currents ranging from 100, 200, 400 and 800 pA generated at −100 mV (**Figure 3**). Current densities of I_LP_ ranged from 0.38 to 171.28 pA/pF, with an average of 36.70+/−2.93 (N = 18). The cause of the change in **I_LP_** in response to changing external conditions, can’t just be related to changes in current flow across a stably-sized leaky patch, because lowered external calcium, increases the apparent inward leaky patch current, even though the driving force for inward calcium ion flow falls dramatically in low compared to standard concentrations of external calcium ions.

**Figure 4:**
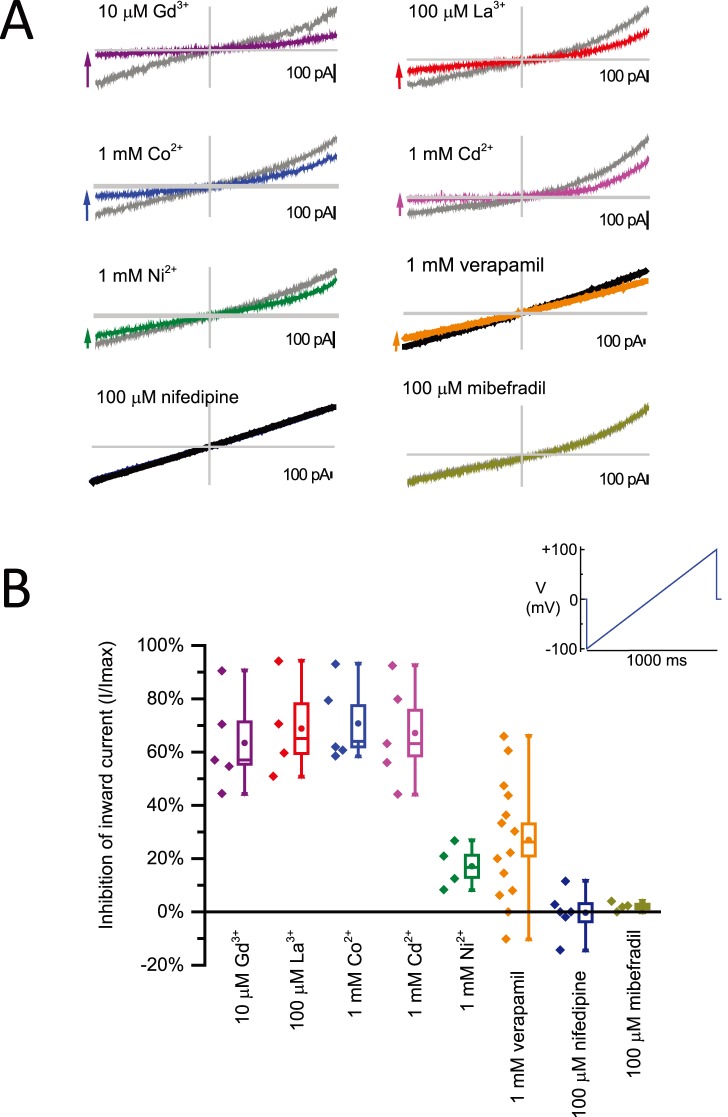
HEK-293T cell currents from a leaky patch (I_LP_) have a characteristic drug blocking profile characteristic of ion channel currents. Bath application of di- and trivalent ions, and calcium channel blockers and their block of I_LP_ during a 1 second voltage ramp. (A) Representative leaky patch currents. Axes cross at zero. X = voltage (mV), Y = current (pA). (B) Data points (left) and box plot (mean +/− SEM, right).

**I_LP_** has a consistent responsiveness to blocking cations and drugs. We evaluated single dose concentrations of the spectrum of divalent and trivalent ions (Gd^3+^, La^3+^, Cd^2+^, Co^2+^, Ni^2+^) and calcium channel blockers (verapamil, mibefradil and nifedipine) that were tested for the specific block of expressed mammalian NALCN currents. 10 µm Gd^3+^, 100 µM La^3+^, 1 mM Cd^2+^ and 1 mM Co^2+^ rapidly block >40% to <90% of the **I_LP_** current (**Figure 4**). 1 mM Ni^2+^ has a consistently lesser effect on the **I_LP_** current (<30%) compared to the other di- and trivalent cations (**Figure 4**). Why 10 µm Gd^3+^ is much more effective at blocking **I_LP_** than 1 mM Ni^2+^, likely relates to the nature of the ion and its capacity to stabilize the membrane patch seal.

Not all externally applied compounds appear to stabilize the leak current. For example, 100 µm mibefradil or 100 µm nifedipine had no observable effect (**Figure 4**). Another calcium channel blocker, verapamil had a highly variable inhibitory effect with a range from zero or negative effect to 2/3 inhibition. The high sensitivity of **I_LP_** to some blocking compounds (eg. 10 µM Gd^3+^, 100 µM La^3+^), but not others (eg. 1 mM Ni^2+^ or 100 µm mibefradil or 100 µm nifedipine) mimics the features of an expressible ion channel with high affinity ligand binding sites for specific drugs.

## Discussion

### Summary of the Leaky Patch Current Characteristics

Here, we describe the consistent and observable features of the leaky patch current, dubbed I_LP_. The observed resistance of a patched cell is the parallel combination of the pipette spanning dome resistance and current leakage between the cell membrane and glass pipette (**see** **Figure 1**). We observe no significant ionic currents after establishing a gigaohm seal in whole cell patches of control HEK-293T cells. We then weakened the tight membrane seals by voltage pulses using the zap function on the patch clamp amplifier, after which we observe large ohmic linear currents after membrane stabilization. If there were a significant contribution of ionic channels within the patch dome, we would have observed native currents during recordings of the gigaseal patch. In the discussion below, we suggest that changes in the leaky patch currents are due to changes to the membrane and glass electrode interface, and we provide a mechanism of how we think this occurs.

Interpretation of the features of the leaky patch current providing in this discussion is based on observations of patch seal biophysics by Fredrick Sachs [20], [26]–[31] and the collaborative effort of Shai Silberberg and Karl Magleby [32], [33].

The observations in this manuscript that require interpretation are the following:

- Leak currents generated by artificial membrane rupture using the ZAP function on the patch clamp amplifier, form a stable resealed membrane with consistent behavior of the leaky patch current derived from a weaker membrane seal due to the “suboptimal” conditions during gigaseal formation of the patch seal.
- The inward sodium leak current was dramatically blocked by replacement of extracellular sodium with large monovalent ion NMDG^+^ on the outside, while there was a significantly lesser NMDG^+^ blocking effect of the outward currents carried in voltage ramp currents generated by outward monovalent ions carried by the Na^+^ or Cs^+^ ions in the intracellular patch pipette. (**Figure 2** **,** **Figure 3**).
- Dropping the extracellular calcium concentration from 1.2 mM to 0.1 mM, resulted in an increase in the outward and inward leak currents. (**Figure 2** **,** **Figure 3**).
- Divalent and trivalent ions are not equivalent in their block of the leaky patch current. **(** **Figure 4**) Gadolinium (Gd^3+^) is the most potent blocker followed by lanthanum (La_3_^+^) which is then followed by Co^2+^, Cd^2+^ and lastly, Ni^2+^. We observed that Ni^2+^ was the weakest cation blocker of the leaky patch current that we tested.
- Standard calcium channel blocking compounds, such as nifedipine and mibefradil were not effective against the leak conductance current. Verapamil, a positively charged calcium channel blocker at physiological pH, had a variable blocking effect, which we interpret partly due its toxicity to HEK cells from their more sickly appearance after drug application of 1 mM concentration. (**Figure 4**).
- Stable leak currents ranged in size from ∼100 pA to as large as 1 nA in a voltage ramp measured at −100 mV, and there was no observable difference in the degree of responses of small or large sized leak currents such as the fraction of blocking effect to di- and tri-valent ion concentrations, or to lowered external calcium concentration or the response to replacing external Na^+^ ions with large monovalent ion, NMDG^+^. (**Figure 2**).

### The Micro-anatomy of the Patch Seal of Living Cells

The ultrastructure of the patch seal (**Figure 1**) has been richly detailed by Frederick Sachs using observations gathered from differential interference contrast light and electron microscopy [20], [28], [30], [31]. Patch seal formation of cells is initiated by the sucking up a sample of the living cortex into the glass pipette. This plug of membrane covered cytoplasm has visible organelles and vesicles and is capable of active contraction within the pipette tip [28], [30], [31]. The pressure of the limited space, delaminates the front end of the migrating membrane cortex, allowing the lipid to bleb and inflate into the pipette, creating a new membrane surface compressed along the walls of the glass electrode with differing composition to the cell surface [28], [30], [31]. The membrane seal is the length of the membrane invaginated within the pipette interior with an upper seal rim that is from tens to a hundred microns above the glass electrode opening (**Figure 1**) [28], [30], [31]. Vesicles may break or fuse to support the patch seal. The newly formed blebs of membrane of the migrating edge are more mobile because they lose some of their cytoskeletal attachments, while others blebs are stressed and restrained as thin filopodia-like extensions of folded membrane roped to an underlying cytoskeleton [28], [30], [31]. Extracellular glycocalyx and protruding extracellular components of membrane ion channels and receptors become compressed and denatured upon the high energy surface of the glass seal surface like fried eggs sticking to a frying pan [20]. The extracellular matrix contribute to a hypertonic, sugary, and viscous solution that can serve as caulking for the developing membrane seal [20]. The membrane center is free from the compression felt by that against the glass rim, and bulges into the pipette, forming a convex “patch dome” under sustained suction that is supported by an underlying cortical cytoskeleton (**Figure 1**), which adapts to its new configuration in the confined space of the pipette interior [20], [28], [30], [31]. The seemingly, endless supply of invaginating lipid enables the patch dome to rise tens of microns into the pipette. The stages of cell stress during patch formation are visible by microscopy, with discrete stages of cell conformity to its confined space in rapidly remodelling cell types like HEK-293T cells [20], [28], [30], [31]. Subdomains within the membrane blebs indicate regions of variable stress. Patch domes can re-shape from angled or irregular-shaped domes, and adopt a more uniform patch dome curvature with increasing suction [20], [28], [30], [31]. It is a cautionary note that treatments designed for studying membrane ion channel function, may have secondary influences on other features of the living cell cortex within the patch pipette.

### Sum of Forces in the Patch Recording of HEK-293T Cells

There are many forces to consider when evaluating the biophysics of the patch seal in HEK-293T cells [20]. These include: A) membrane tension from the hydrostatic fluid pressure within the pipette; B) Van der Waals attractive forces between the patch pipette glass and membrane; C) electro-osmotic forces that generates a streaming lubrication of cations at the glass-membrane interface which contributes to a pH and voltage-dependent creep of membrane up the pipette walls; D) the viscoelasticity of the cytoplasm and the viscosity of the liquid components of the membrane; E) the cytoskeleton which has pulling and pushing forces on the patch seal and patch dome. The cytoskeleton may stiffen with changes in intracellular calcium concentrations, or in response to hypertonicity induced changes. High osmolarity recruits membrane stiffening cross-linking proteins between the plasma membrane and actin cytoskeleton such as ezrin, radixin or moesin [34]. The potential contribution of the cytoskeleton will depend on the integrity of the cell cortex under the patch, which is greatest for on-cell patch, and least for the excised membranes of the inside-out or outside-out patch configurations. The cytoskeleton can serve as a porous plug obstructing fluid flow in attached cells, exacerbating transmural pressure gradients. Strain kinetics from an attached cytoskeleton slows the viscoelastic relaxation ∼5 fold for cell-attached patches compared to inside-out patches during patch seal formation [20].

### Understanding of the Apparent Contradiction of a Stable Membrane Seal, in the Presence of a Variably Sized Leak Current

The phenomenal adherence of membrane onto glass is behind the wizardry of the patch clamp technique introduced by Erwin Neher and Bert Sakmann [35]. This technique allows for the real time measurement of the tiny currents through ionic channel and receptors across living patches of cell membranes. The seal adhesive force is reported to be approximately ∼1.6 mN/m (or J/m^2^) for HEK-293T cells [20] and approximately the same or higher for artificial membrane bilayers [36], regardless of conditions of a cell attached patch. The glue holding the membrane glass appears largely fixed, even under the assault of ripped membrane patches from the rest of the cell (inside-out patch) or membrane rupture by excessive suction or electrical “ZAP” pulses (whole cell configuration) [12]. The membrane patch can be pulled away from the electrode in the whole cell configuration and the bleb of membrane can rapidly reform as a convex surface on the end of the electrode, re-establishing its strong adherence (outside-out patch) [12]. The membrane glass is predicted to be very close (<1–2 nm) to account for the impenetrability of substances across a gigaohm seal [20]. The membrane gigaseal is impenetrable for substances attempting to escape the sealed patch membrane, such as membrane permeabilizing agents like nystatin. These permeabilizing agents are limited to the area bathed by intracellular solution under the patch electrode [37]. The membrane seal is also a barricade for substances attempting to enter under a cell attached patch, such as externally perfused drugs entering the isolated patch of membrane under the glass electrode [38].

The primary adhesive force for the membrane gigaseal are assumed to be Van der Waals attraction forces, the intermolecular forces underlying the adhesion of sticky tape or Geckos sticking to ceilings, named after Johannes Diderik van der Waals [39]. Van der Waals forces are accompanied by electrostatic interactions of ions within the patch seal. Membrane and glass have a low dielectric constant, which is a measure of the permittivity of the material to serve as an insulator of electrical charge. The thickness of the membrane seal is less than the Debye length in the bath, the Debye length being the radial sphere of influence that a charge carrier’s net electrostatic effect persists in solution, outside of which electrical charges are effectively screened and neutralized. Both the membrane and glass are negatively-charged charged surfaces, which would be repulsed from each other without the Van der Waals attractive forces attractive force keeping the two surfaces together. The negatively-charged space inbetween the two surfaces necessarily excludes anions and attracts cations. Voltage gradients established from the recording pipette to bath generate the flow of these mobile cations which then drag water into this space between the membrane and glass. The generation of the electro-osmotic force ensure there is always a dynamic, lubricating cationic solution flowing between the glass and membrane within the patch seal (**Figure 1**). The movement of the lubricating fluid is the motor driving the continuous and visible patch creep of lipid membrane in the recording pipette at a rate of 0.1 to 1 microns per minute, even in the absence of suction (**Figure 1**) [20], [32]. The area of adhesion to glass that contributes to the seal resistance is a dynamically creeping annulus of membrane along the patch pipette walls, whose upper rim can range from 10 to 50 um to as high as 100 um at the uppermost along the sides of the pipette walls [20], [32]. The adhesive force of Van der Waals attractive forces ensures a primary stickiness of the annulus of membrane to glass for the membrane seal, while electro-osmotic forces ensures that there is always a lubricating cationic solution serving as a conduit for variable-sized, but stable leak currents between the glass and membrane surfaces of the membrane seal.

### Sodium Leak Currents Generated in Patch Seals Can Be Replicated in Cell Membranes, Artificial Lipids and Silicone Rubber

The biophysical forces that contribute to the tight membrane seal and the leaky patch currents, can be replicated without a cell substrate like HEK-293T cells. The same features are found with glass pipettes and artificial membrane layer of phospholipids like phosphphatidylcholine [33]. And further, it isn’t even a requirement to have an organic medium at all to observe leak conductance currents in patch seals. Lipid membrane effects can be replicated with a non-biological polymer, such as Dow Chemical’s Sylgard. Sylgard is a hydrophobic silicone rubber used to coat the sides of micro-electrode tips for electrophysiology [19]. Sylgard coating reduce trans-glass electrode capacitance or can serve as a rubber substrate for cell culture dishes and pinning animal and tissue specimens to dissecting pads. Glass on Sylgard forms gigaohm seals and “rubber currents” with single channel, and binary opening - closing gating events, with a discrete pore size, circumscribed for its selective permeability for particular cations over anions [29]. Sylgard recordings have a permeability hierarchy of cations from most to least permeable ion based on reversal potentials, where Na^+^ is the most permeant ion over K^+^ > Cs^+^ > Li^+^ > Guanidinium^+^ > H^+^ > Mg^2+^ > Sr^2+^ > Ca^2+^ > Tris^+^ > Ba^+^ > Mn^2+^ > Arginine^+^ > La^3+^ > Acetylcholine^+^ [29]. We assume that most of the observations for the sodium leak currents in cells described in this manuscript could be replicated with patch seals onto artificial lipids and Sylgard silicone rubber where the same physical forces generate a cationic selective crevice between the glass and substrate.

### The Blocking Effect of the Sodium Leak Current by Cations and NMDG^+^ Is Related to Electrostatic Forces and the Electro-osmotic Flow of Mobile Cations through Membrane Seal Crevices

To apply di- and tri-valent ions to the outside medium is to titrate positive-charges into the membrane-glass seal space and neutralize the electrostatic repulsion of the negatively-charged glass and membrane, effectively increasing the seal resistance, and blocking the leak conductance through virtual ion channel protein-gated currents. The most effective blocking ion that we tested was gadolinium (Gd^3+^) which has the highest charged density compared to other trivalent cations that we tested such as lanthanum (La^3+^) or divalent ions (Co^2+^, Cd^2+^ and Ni^2+^). Another means to lower the membrane-glass electrostatic repulsion is to replace the recording glass electrode with a glass electrode with a less negative surface charge [20]. Soda lime generates half the “rubber currents” in Sylgard compared to similar Sylgard recordings with borosilicate glass electrodes [29]. Borosilicate glass possess approximately double the negative surface charge of soda lime glass and so the response to changes in glass is a reflection of its electrostatic influences on the cations within the membrane seal [20]. Changing the charge within the membrane seal is to alter the electro-omotic water and ion flow through the seal which translates into a vector displacement (direction and velocity) of the patch creep of the adhesive sealed membrane along the walls of the pipette [20], [32]. At pH of 7, the patch migrates upward for negative pipette potentials, and reversed polarity and migrates downward inside the pipette when positive voltage gradients are imposed [20], [32]. The motor driving the patch creep is the underlying movement of the mobile cations within the membrane seal [20]. Upward displacement of the patch creep is faster for pH 9 than pH 7, and reverses direction and accelerates downward as the pH drops to 5 and below [20]. The more acidic the conditions, the greater the spreading of lipid on glass [40], and the tighter and faster is the formation of the membrane patch seal [33]. As expected soda lime glass with lowered surface charge, dramatically reduces the voltage-dependent creep compared to patch seals with borosilicate glass electrodes [20]. Substituting less mobile cations, such as NMDG^+^ into the seal space is to reduce the electro-osmotic flow of inward currents, and slows the rate of voltage-dependent creep of the patch within the pipette. The slowing of the inward electro-osmotic flow of ions would explain our observations of the reduced inward leak currents when external NMDG^+^ replaced external Na^+^ ions.

### Changing Calcium Ion Concentration is to Change the Total Adhesive Force between Glass and Membrane Surfaces

Calcium ions directly influence the adhesive force between glass and membrane surfaces. Force changes of membrane seals are quantifiable by atomic force microscopy (AFM) where the cantilever probe is coated with cells or artificial lipids and the force displacement is measured as the cantilever probe is lowered onto glass surfaces [33]. Increasing Ca^2+^ concentration from 10^−8^ to 10^−1^ increases the mean adhesive force onto glass ∼2.6 fold for HEK-293T cell membranes, and increased ∼1.8 fold for artificial membranes composed of phosphatidylcholine [33]. Similar increases in adhesion force in the presence of increasing calcium concentrations was observed for other artificial membranes composed of phosphatidylinositol or phosphatidylserine [33]. Mg^2+^ can replace Ca^2+^ in driving increases in adhesion forces between glass and lipid [33]. Adhesive force changes are not related to the influence of calcium’s or magnesium’s ionic strength. Increasing Na^+^ concentration from 10^−4^ to 10^−1^ for example, as no influence on the adhesive strength of membrane seals as measured by atomic force microscopy [33]. Increasing external calcium concentration decreases the time to gigaseal formation with a magnitude of suction of 0.3 kPa [33]. If the concentration of calcium drops to <10^−8^, the adhesive forces can be very weak and tight seals are not possible despite increasing suction of membrane into the pipette [33]. We observed that lowering calcium from 1.2 mM external calcium to 0.1 mM calcium results in an increase in sodium leaks currents through virtual leak conducting channels. This can be explained by the decrease in adhesion force of the membrane-glass interface when calcium concentrations are lowered.

### A Similar Virtual Pore Size of the Sodium Leak Currents Even If Generated by Artificial Membrane Rupture

The block by di- and tri- valent cations at particular doses and sensitivity to NMDG^+^ suggest leaky patch currents permeate through a discrete pore size, even with variations in total leak currents which can differ by more than tenfold (100 pA to 1 nA, measured as the size of current at −100 mV). The differing leak current sizes may be a reflection of the lengths of the crevices within the glass-membrane seal, which serves as the effective length of a channel pore for the electro-osmotic flow of ionic current between the extracellular space and the pipette interior. Also affecting the rate of flow of leak current is the number of available channel crevices of a discrete pore size in the membrane-glass seal interface. Crevices in the membrane seals of HEK cells likely possess the same discrete, single channel gating behavior that is observable for the sodium leak currents generated with non-biological substrates like glass-Sylgard seals [29]. The sum of forces such as Van der Waals and electrostatic interactions between glass and cell membrane, or between glass-lipid or glass-silicone rubber, must equivalently shape the size of the crevice pore within particular limits for the flow of the lubricating stream of cations that permeate the patch glass seal interface.

Artificial membrane rupture by voltage zap creates artificial holes into the patch dome and facilitate a whole cell configuration where the intracellular pipette fluid is then continuous with the intracellular environment through these holes [26]. Artificial membrane rupture also disturbs the membrane seal adhesion to the glass electrode, which quickly settles to a new equilibrium after membrane resealing and stabilization, because of the strong attraction of lipid to glass [26]. At this new equilibrium, more crevices, (and perhaps shorter ones) likely appear of discrete sizes tunneling in the interface between the membrane and patch glass, generating a larger observable leak current size. At a stable leak current, Van der Waals attraction forces tolerates a particular leak capacity through the membrane seal crevices. The equilibrium maintaining the size of leak current is disturbed after the perfusion of di-, trivalent cations, Ca^2+^ or NMDG^+^ containing extracellular solutions. These extracellular treatments can alter the adhesive energy and mobilize the ions in the crevices, necessarily selective for cations by the membrane-seal interface lined with negative charges. Changes to the extracellular solutions surrounding the seal drives changes to the electro-osmotic flow of cations in the membrane-glass seal, enhancing or depressing leak currents that have the illusory properties of those mimicking ion channel protein conductances.

## Conclusions: Leaky Patch Currents Behave as Compelling Ion Channel Conductances

Here, we have characterized the ion currents generated by the leaky patch seal. Our investigation began when we were trying to interpret the leak conductances expected from expressible snail NALCN cDNA splice variants, with selectivity filters that resembling calcium (EEEE) and sodium (EKEE) channels [13], [14]. We also wanted to replicate the features of the reported sodium leak currents observed from expressible human NALCN channels [15]–[17]. Currents through a leaky patch are compelling ion currents that have attributes of ion channel conductances, and display consistent behavior like expressible channel proteins, with a particular drug blocking profile. We propose that channel crevices in membrane seals serve as aqueous pathways through the hydrophobic lipid environment formed along the membrane seal of the pipette. These are sodium leak conduits and are not anionic (chloride channels) because they possess a selectivity filter lined by the negative-charges of the membrane and glass interface. These leak currents are not calcium-selective because calcium directly interferes with the membrane-glass adherence. A variable number of channel crevices appear in the membrane-glass interface, and these are necessarily filled with a lubricating stream of cations attracted to the negatively-lined, glass-membrane interface. We can assume that Gd^3+^ and calcium sensitive sodium leak conductance accompanies all weak patch seals (including Sylgard rubber seals). There is a phenomenal adherence of lipid to glass which electrophysiologists exploit to carry out recording gymnastics of patches of cell membrane. And this lipid to glass adherence appears fixed up to a point, where it can accommodate the sodium leak as a drip or a gushing leak across the membrane seal, until the dam (i.e. membrane seal) completely breaks. It is at this point of seal breakage that an electrophysiologist will get a new pipette and cell to record. Signals from leaky recordings can be resolved from the non-specific and linear leak component of the aggregate waveform. Here, we have attempted to define the features of this usually discounted, leak conductance through weak patch seals, as a baseline on which to evaluate sodium leak currents from expressible ion channels, such as NALCN leak conductance channel.
